# Effect of Cotton Leafroll Dwarf Virus on Physiological Processes and Yield of Individual Cotton Plants

**DOI:** 10.3389/fpls.2021.734386

**Published:** 2021-10-01

**Authors:** Ved Parkash, Divya Bhanu Sharma, John Snider, Sudeep Bag, Phillip Roberts, Afsha Tabassum, Dalton West, Sameer Khanal, Nelson Suassuna, Peng Chee

**Affiliations:** ^1^Department of Crop and Soil Sciences, University of Georgia, Tifton, GA, United States; ^2^Institute of Plant Breeding, Genetics, and Genomics, University of Georgia, Tifton, GA, United States; ^3^Department of Plant Pathology, University of Georgia, Tifton, GA, United States; ^4^Department of Entomology, University of Georgia, Tifton, GA, United States

**Keywords:** photosynthesis rate, stomatal conductance, electron transport, leaf temperature, cotton leafroll dwarf virus

## Abstract

Cotton leafroll dwarf disease (CLRDD) caused by cotton leafroll dwarf virus (CLRDV) is an emerging threat to cotton production in the United States. The disease was first reported in Alabama in 2017 and subsequently has been reported in 10 other cotton producing states in the United States, including Georgia. A field study was conducted at field sites near Tifton, Georgia in 2019 and 2020 to evaluate leaf gas exchange, chlorophyll fluorescence, and leaf temperature responses for a symptomatic cultivar (diseased plants observed at regular frequency) at multiple stages of disease progression and for asymptomatic cultivars (0% disease incidence observed). Disease-induced reductions in net photosynthetic rate (*A*_n_, decreased by 63–101%), stomatal conductance (*g*_s_, decreased by 65–99%), and efficiency of the thylakoid reactions (32–92% decline in primary photochemistry) were observed, whereas leaf temperature significantly increased by 0.5–3.8°C at advanced stages of the disease. Net photosynthesis was substantially more sensitive to disease-induced declines in *g*_s_ than the thylakoid reactions. Symptomatic plants with more advanced disease stages remained stunted throughout the growing season, and yield was reduced by 99% by CLRDD due to reductions in boll number per plant and declines in boll mass resulting from fewer seeds per boll. Asymptomatic cultivars exhibited more conservative gas exchange responses than apparently healthy plants of the symptomatic cultivar but were less productive. Overall, it is concluded that CLRDV limits stomatal conductance and photosynthetic activity of individual leaves, causing substantial declines in productivity for individual plants. Future studies should evaluate the physiological contributors to genotypic variation in disease tolerance under controlled conditions.

## Introduction

Cotton leafroll dwarf virus (CLRDV; family Solemoviridae, genus *Polerovirus*) is known to cause cotton blue disease (CBD), and its occurrence was first reported in Africa in 1949, followed by reports of the disease in Asia and South America (Fang et al., 2010). Cotton yield losses up to 80% have been observed from CLRDV in South America (Silva et al., 2008), and the virus is now an emerging threat to cotton production in the United States (Avelar et al., 2019). CLRDV was first reported in Alabama in 2017 (Avelar et al., 2019) and has subsequently been documented in 10 other cotton producing states, with disease incidence varying from less than 1% to more than 20% across the United States cotton belt (Aboughanem-Sabanadzovic et al., 2019; Tabassum et al., 2019; Alabi et al., 2020; Ali et al., 2020; Ali and Mokhtari, 2020; Faske et al., 2020; Iriarte et al., 2020; Price et al., 2020; Thiessen et al., 2020; Wang et al., 2020). Interestingly, cotton plants infected with the United States strains of CLRDV showed different symptoms from CBD; therefore, the disease caused by this virus has been named as cotton leafroll dwarf disease (CLRDD; Brown et al., 2019). Numerous symptoms have been associated with CLRDD. A few of the notable symptoms include reddening of leaves and petioles, leaf wilting, subsequent drooping, crinkling, and deformation of the leaves above the nodes, which had reddened leaves, development of intense green foliage, and shortening of upper internodes resulting in stunted plants (Tabassum et al., 2019, 2020; Bag et al., 2021). Severe disease infection could result in reduced boll set and lower seed-cotton yield per plant (Avelar et al., 2020). The disease symptomology can vary substantially depending upon plant developmental stage at the time of infection, stage of disease progression, location, and variety.

CLRDV is transmitted by the cotton aphid (*Aphis gossypii* L.; Avelar et al., 2020) in a circulative and persistent manner (Silva et al., 2008). CLRDV is a positive sense, single-stranded RNA virus, and it is a phloem-limited virus, meaning it replicates and circulates mainly in the phloem tissue of the host plant (Silva et al., 2008; Jiménez et al., 2021). In the United States, the partial genome sequence of CLRDV was first sequenced from isolates collected from Alabama (Avelar et al., 2020), and the complete genome was subsequently completed from an isolate collected in Georgia (Tabassum et al., 2020, 2021). The genomic sequence of the CLRDV isolate from Georgia was between 95 and 98% identical to the genome of other CLRDV isolates reported in the United States (Alabama, GenBank accession number MN071395; Texas, MN872302) and South America (KF359947, KF906261, KF906260, NC_014545, GU167940, and HQ827780; Tabassum et al., 2020, 2021). Despite obvious genomic similarity, there are three unique genotypes of CLRDV recognized globally: “atypical,” “typical,” and “CLRDV-US” (Avelar et al., 2020; Iriarte et al., 2020).

Since CLRDD is new to the United States, there is very little information available on the physiological response of cotton to the disease. With other viral plant diseases that visibly affect foliage, significant reductions in net photosynthesis (*A*_n_) have been documented (Ryšlavá et al., 2003). Furthermore, virus-induced declines in *A*_n_ have been associated with reduced efficiency of the light dependent processes of the thylakoid reactions, lower stomatal or mesophyll conductance to CO_2_, or reduced activity of the carbon fixation reactions of the Calvin cycle (Sampol et al., 2003; Moutinho-Pereira et al., 2012; Souza et al., 2017). Another important observation from the previously noted studies is that the degree of photosynthetic inhibition observed and the primary factor contributing to photosynthetic decline can vary substantially depending on plant species, cultivar, and virus. Because CLRDV causes visible changes in leaf coloration and turgor, it is reasonable to assume that CLRDV-induced photosynthetic decline might be associated with reductions in stomatal conductance (*g*_s_) and possibly the activity of the thylakoid reactions.

During the 2019 and 2020 growing seasons, experiments containing multiple cotton cultivars were conducted at field sites in Tifton, GA. In both seasons, plants with CLRDD symptoms of varying severity were consistently observed in the early season for cotton cultivar DG 3615 B3XF (symptomatic cultivar, *S*), whereas other cultivars at the same sites exhibited 0% disease incidence (asymptomatic cultivars, *A*). Since some cotton cultivars have recently been shown to differ in their stomatal response to vapor pressure deficit (Shekoofa et al., 2021), otherwise normal plants of *A* and *S* cultivars may exhibit inherent differences in gas exchange responses under the same field conditions. It was hypothesized that diseased plants of the symptomatic cultivar would exhibit lower light-saturated photosynthetic rates, reduced photochemical efficiencies of photosystem II, declines in stomatal conductance, increased leaf temperature, and reduced productivity relative to plants of the same cultivar without conspicuous disease symptoms. We also hypothesized that asymptomatic cultivars would exhibit more conservative gas exchange responses (lower *g*_s_) than otherwise normal plants of the symptomatic cultivar under field conditions. Thus, the objectives of the current study were to determine gas exchange, chlorophyll fluorescence, leaf temperature, and yield responses for a susceptible cultivar at multiple stages of disease progression and for otherwise healthy-appearing plants of symptomatic and asymptomatic cultivars.

## Materials and Methods

### Experimental Details

**Figure 1:**
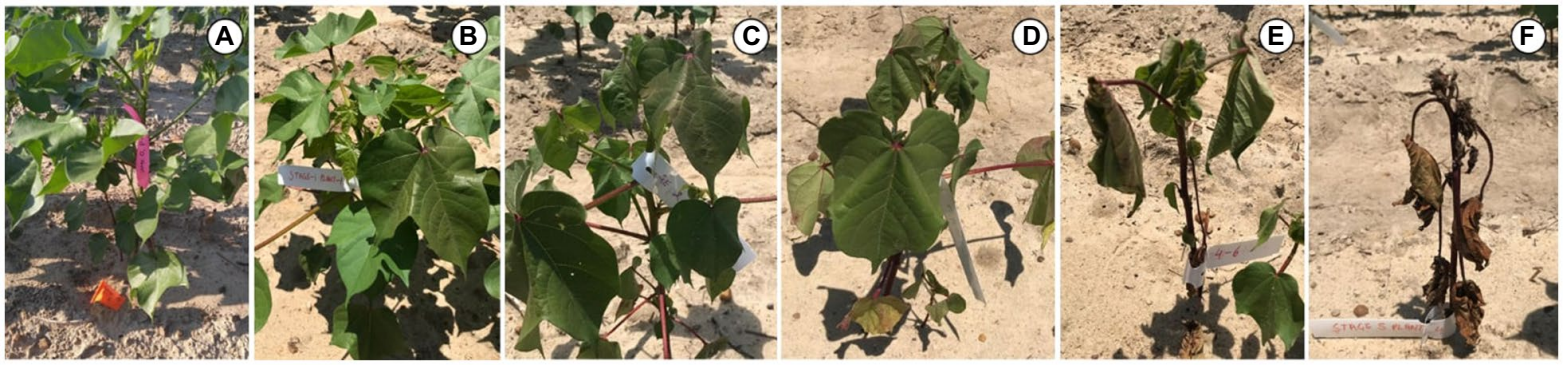
Images showing symptomology associated with various stages of CLRDD progression in *Gossypium hirsutum* cv. DG 3615 B3XF. **(A)**=*S*_0_ (healthy plants of the symptomatic variety), **(B)**=*S*_1_ (initial reddening, but no wilting), **(C)**=*S*_2_ (reddening plus initial leaf droop), **(D)**=*S*_3_ (loss of leaf turgor), **(E)**=*S*_4_ (severe wilt and advanced leaf chlorosis), and **(F)**=*S*_5_ (senescent plant).

Experiments to evaluate the physiological response of cotton to CLRDD were conducted in two different growing seasons at field sites near Tifton, Georgia, United States. Specifically, cotton variety trials with cultivars arranged in a randomized complete block design, were conducted on University of Georgia research farms during the 2019 and 2020 growing seasons. The soil at both locations is a Tifton sandy loam, and the crop was planted on June 17, 2019 and May 11, 2020. Cotton plants typically produce floral buds for approximately 3-week period prior to flowering, referred to as “squaring.” In both seasons, disease symptoms were present in plants near the early squaring stage of development, where as much as 20–25% of the plants in a particular variety (DG 3615 B3XF; Dyna-Gro® Seed) showed symptoms of CLRDD. This particular variety was considered as symptomatic, where affected plants went through a series of five predictable stages as the disease became progressively more severe. The stages are depicted in Figure 1, and they include *S*_0_ (healthy plants of the symptomatic variety), *S*_1_ (initial reddening, but no wilting), *S*_2_ (reddening plus initial leaf droop), *S*_3_ (loss of leaf turgor), *S*_4_ (severe wilt and advanced leaf chlorosis), and *S*_5_ (senescent plant). Other varieties in the experiment showed no symptomatic plants (0% disease incidence), suggesting that they are not susceptible to the disease. These cultivars included ST 5600 B2XF and DP 1851 B3XF in 2019 and ST 5122 GLT in 2020. For simplicity, these cultivars are referred to as asymptomatic (A) cultivars.

Physiological assessments were conducted on asymptomatic cultivars and on *S*_0_ through *S*_4_ in 2019 and *S*_0_ through *S*_3_ in 2020. These assessments were conducted immediately following stage identification and plant tagging (July 9 in 2019 and July 27 in 2020) and approximately 3weeks later, once plants had time to recover and produce new leaves (August 4, 2019 and August 16, 2020). The experiment was treated as a completely randomized design with individual plants being treated as units of replication. The number of replications per cultivar/stage ranged from 5 to 10.

### Virus Detection

Petioles and leaf tissues from symptomatic plants with different stages (*S*_0_–*S*_5_) of disease symptoms were collected for the detection of CLRDV. Five asymptomatic leaf and petiole tissues were also collected from other cultivars for virus detection. The detection protocol used for CLRDV was conducted using a two-step reverse-transcriptase polymerase reaction (RT-PCR) according to the methods given in Tabassum et al. (2020). Total RNA was extracted using the modified CTAB method (Sharman et al., 2015). Complementary DNA (cDNA) was synthesized from 2.5μg of total RNA using Superscript III reverse transcriptase (Invitrogen, United States) and specific reverse primers targeting ORF 3. The cDNA was used for PCR with primers SB11F (5' AGG TTT TCT GGT AGC AGT ACC AAT ATC AAC GTT A 3') and SB11R (5' TAT CTT GCA TTG TGG ATT TCC CTC ATA A3') to amplify the 803bp fragment spanning ORF 3 and ORF 4, encoding virus coat protein (P3) and movement protein (P4) genes. The amplified products were analyzed in 0.8% agarose gel electrophoresis with 1% TAE buffer. Amplicons of the expected size (~803bp) were gel-purified, cloned into the pGEM-T easy cloning vector (Promega, United States), and sequenced using SP_6_-T_7_ sequencing primers (GenScript, United States).

### Gas Exchange and Chlorophyll Fluorescence Measurements

For single leaf measurements, an LI-6800 and an LI-6400XT were used during the 2019 and 2020 growing seasons, respectively. Both instruments are open gas exchange systems with the ability to obtain gas exchange and fluorescence measurements simultaneously over the same leaf area. All measurements were conducted on the uppermost, fully expanded leaf on each plant between 12:00 and 15:00h, with leaf tissue being sealed in the chamber until steady-state photosynthesis measurements were obtained (60–120s). Leaf chamber settings for both instruments at the time of measurement include a flow rate of 600μmols^−1^, reference [CO_2_]=400μmolmol^−1^, photosynthetically active radiation (PAR)=1,500μmolm^−2^ s^−1^, chamber air temperature=ambient air temperature at the time of measurement, relative humidity=ambient humidity (between 50 and 70%). Gas exchange parameters recorded at that time included net photosynthesis (*A*_n_) and stomatal conductance to water vapor (*g*_s_). Leaf temperature (*T*_leaf_) was recorded simultaneously, along with air temperature at the time of measurement (*T*_air_), and the leaf to air temperature differential (*T*_leaf_−*T*_air_). Table 1 shows weather conditions prevailing during seven day period proceeding the date of physiological measurements. Chlorophyll fluorescence assessments were also conducted at the same time using the methods and calculations discussed extensively elsewhere (Earl and Ennahli, 2004; Chastain et al., 2016; Meeks et al., 2019; Virk et al., 2020b). The primary fluorescence parameters of interest included actual quantum yield of photosystem II (*Φ*_PSII_) and rate of electron flux through photosystem II (ETR).

**Table 1:** Daily maximum air temperature (*T*_max_), minimum air temperature (*T*_min_), average temperature (*T*_avg_), daily maximum air vapor pressure deficit (VPD), and average cumulative daily solar radiation for a 7-day period preceding the date of physiological assessments.

Date	*T*_max_ (°C)	*T*_min_ (°C)	*T*_avg_ (°C)	VPD (kPa)	Total solar radiation MJ m^−2^
July 27, 2019	32.67	19.06	25.48	2.67	21.88
August 16, 2019	35.15	23.46	28.22	1.63	20.25
July 9, 2020	33.03	21.27	25.81	2.92	19.17
August 4, 2020	34.9	21.85	26.64	2.22	19.01

### Diurnal Leaf Temperature Response

While the physiological assessments conducted in the current study provide valuable insight into the response of cotton to CLRDV, they are inherently time-consuming. This limits their ability to capture diurnal trends with high temporal resolution. Because leaf temperature was consistently higher in plants exhibiting conspicuous disease symptomology, the follow up experiment was conducted to evaluate the diurnal temperature responses of individual leaves at different stages of disease progression for the symptomatic cultivar. Specifically, five plants were measured at disease stages from S_0_ to S_3_ on July 24, 2020. Measurements were conducted on uppermost, fully expanded leaves at 6:00am, 8:00am, 10:00am, 12:00pm, 2:00pm, 4:00pm, 6:00pm, and 8:00pm; leaf temperature was obtained using an infrared thermometer (Fisherbrand™ Traceable™ Infrared Thermometer). Concomitant with leaf temperature measurements, weather data were collected in 15min intervals on the day of sampling using an on-site weather station. Diurnal trends in air temperature, solar radiation, and leaf to air temperature difference are reported herein.

### Per Plant Yield Components

Yield data were not initially obtained for the 2019 season, primarily because stunted plants exhibited boll set levels near zero, and it was initially assumed that there would be little benefit to determining lint yield in this season. However, an effort was made to quantify per plant lint yield and yield component responses for each sample during the 2020 season. Each plant was hand harvested at crop maturity, and the total number of bolls was recorded. Thereafter, fiber was separated from seed using a small, table-top saw gin, and lint percent and seed index were recorded. From these data, boll number per plant, boll mass, seed number per boll, and lint weight per seed were determined.

### Statistical Analysis

Prior to statistical analysis, samples were assigned to specific treatments. In 2019, treatments included *A*_1_, *A*_2_, *S*_0_, *S*_1_, *S*_2_, *S*_3_, and *S*_4_; in 2020, treatments included *A*, *S*_0_, *S*_1_, *S*_2_, and *S*_3_. The effect of treatment on mid-day physiological parameters of interest and yield components was assessed using a one-way analysis of variance within each sample date and year. *Post hoc* means separation was performed using Fisher’s protected LSD analysis. For the diurnal study, a similar analysis was conducted to assess for the effect of disease stage on leaf temperature within each diurnal sample time.

The underlying processes driving photosynthesis may exhibit different sensitivities to wilt-inducing stress, as has been observed under drought conditions for field-grown cotton (Chastain et al., 2014; Snider et al., 2014). Medrano et al. (2002) further suggested that the sensitivity of photosynthesis or photosynthetic component processes could be defined by plotting the parameter of interest vs. *g*_s_, which is used as a reference indicator of stress. Thereafter, hyperbolic functions can be fit to the data, and specific threshold *g*_s_ values for a given process used as an indicator of tolerance. If two processes differ in the *g*_s_ value needed to reach the same percent decline, the two processes differ in stress tolerance. In the current study, *A*_n_ and ETR were plotted vs. *g*_s_ for data collected over two growing seasons, on the susceptible cultivar only, and on the day that plants were initially staged. Hyperbolic functions were fit to these data, and the *g*_s_ value required to cause 50% decline from the maximum observed value was estimated *via* interpolation for *A*_n_ and ETR.

## Results

### Virus Detection

Petioles and leaf tissues were collected from the symptomatic and asymptomatic plants to confirm the presence of CLRDV. Plants of the symptomatic cultivar (DG 3615), ranging from stage 0 to stage 5 of CLRDD progression, were tested for the presence of CLRDV in both years of the experiment. In the 2019 and 2020 seasons, using end-point RT-PCR, the presence of CLRDV was confirmed at all the stages of symptom development by amplification of the ORF 3 and ORF 4 encoding coat protein (P3) and movement protein (P4), respectively. The amplicon of ~800bp was amplified and sequenced. Nucleotide sequences of ORF 3 and ORF 4 are 93–100% identical with other gene sequences available in NCBI GenBank, confirming the presence of the virus. CLRDV was also detected from a high percentage of asymptomatic plants of all other cultivars assessed (data not shown; DG3615, ST 5600 B2XF and DP 1851 B3XF in 2019 and ST 5122 GLT in 2020). The near-complete sequence of the CLRDV genome was generated from these symptomatic plant tissues and submitted to NCBI GenBank (MT800932) as part of a concurrent research effort (Tabassum et al., 2021).

### Disease Symptoms

A particular disease progression was consistently associated with CLRDD during the early season just prior to floral bud development or “squaring.” In particular, plants went through a series of predictable stages, initially exhibiting a slight reddening of leaf tissue followed by progressive declines in tissue turgor, increases in leaf wilting and chlorosis of the shoot apex, and eventually, death of plant foliage (Figure 1). The progression from conspicuous disease onset to complete defoliation was as little as 5–7days. Interestingly, some senesced plants would sprout new, green leaf tissue, but they remained stunted throughout the season relative to neighboring plants, with little to no harvestable cotton (personal observation).

### Gas Exchange and Chlorophyll Fluorescence Parameters

**Figure 2:**
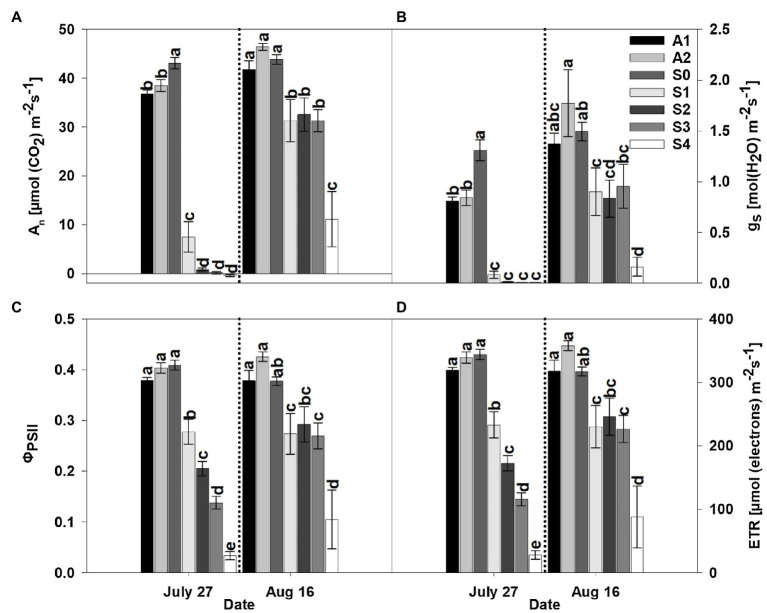
Net photosynthesis rate (*A*_n_; **A**), stomatal conductance (*g*_s_; **B**), actual quantum yield of photosystem II (*Φ*_PSII_; **C**), and rate of electron flux through photosystem II (ETR; **D**) for asymptomatic cultivars (*A*_1_ and *A*_2_) and different stages of disease progression in symptomatic cultivars (*S*_0_ through *S*_4_) measured at two different dates in 2019. Error bars indicate standard errors of the mean. *Post hoc* means separation was performed using Fisher’s protected LSD analysis. Within each measurement date, bars sharing a common letter are not significantly different at *p*≤0.05.

**Figure 3:**
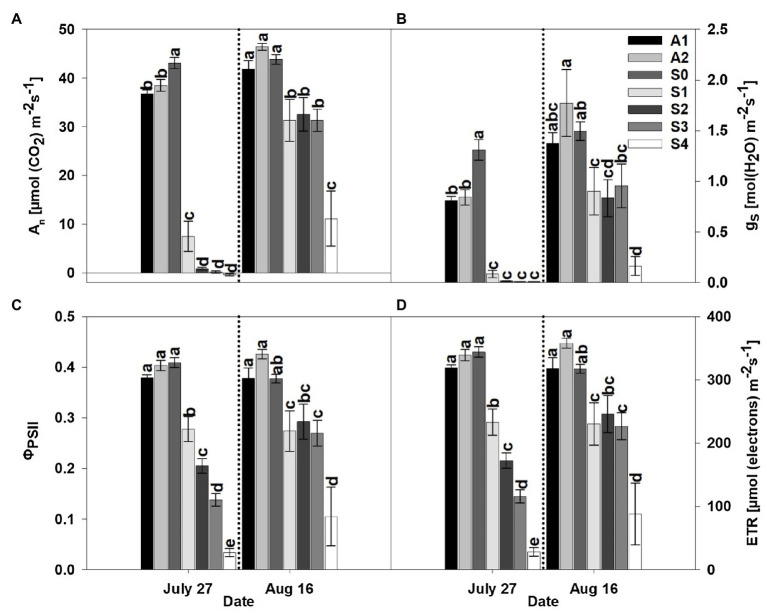
Net photosynthesis rate (*A*_n_; **A**), stomatal conductance (*g*_s_; **B**), actual quantum yield of photosystem II (*Φ*_PSII_; **C**), and rate of electron flux through photosystem II (ETR; **D**) for an asymptomatic cultivar (A) and different stages of disease progression in symptomatic cultivars (*S*_0_ through *S*_3_) measured at two different dates in 2020. Error bars indicate standard errors of the mean. *Post hoc* means separation was performed using Fisher’s protected LSD analysis. Within each measurement date bars sharing a common letter are not significantly different at *p*≤0.05.

The parameters *A*_n_, *g*_s_, *Φ*_PSII_, and ETR were significantly affected by treatment. Specifically, CLRDD caused substantial reductions in *A*_n_, *g*_s_, *Φ*_PSII_, and ETR as the disease progressed to successively more advanced stages (Figures 2, 3). For example, on the initial measurement date, *A*_n_ ranged from 83% lower for *S*_1_ (7.5μmolm^−2^ s^−1^) to 101% lower for *S*_4_ (−0.4μmolm^−2^ s^−1^) in 2019 and 63% for *S*_1_ (14.9μmolm^−2^ s^−1^) to 97% for *S*_3_ (1.2μmolm^−2^ s^−1^) lower in 2020, when compared with *S*_0_ (43.1μmolm^−2^ s^−1^ in 2019 and 40.4μmolm^−2^ s^−1^ in 2020). Similarly, *g*_s_ decreased 94% for *S*_1_ (0.08molm^−2^ s^−1^) to 99% for S_4_ (0.01molm^−2^ s^−1^) in 2019 and 65% for *S*_1_ (0.24molm^−2^ s^−1^) to 97% for *S*_3_ (0.02molm^−2^ s^−1^), in 2020, relative to *S*_0_ (1.31molm^−2^ s^−1^ in 2019 and 0.70molm^−2^ s^−1^ in 2020). Declines in *Φ*_PSII_ and ETR for diseased plants relative to *S*_0_ (*Φ*_PSII_=0.41 and ETR=344μmolm^−2^ s^−1^ in 2019 and *Φ*_PSII_=0.45 and ETR=297μmolm^−2^ s^−1^ in 2020) plants ranged from 32% for *S*_1_ (*Φ*_PSII_=0.28 and ETR=233μmolm^−2^ s^−1^) to 92% for *S*_4_ (*Φ*_PSII_=0.03 and ETR=28μmolm^−2^ s^−1^) in 2019 and from 35% for *S*_1_ (*Φ*_PSII_=0.30 and ETR=194μmolm^−2^ s^−1^) to 56% for *S*_3_ (*Φ*_PSII_=0.20 and ETR=129μmolm^−2^ s^−1^) in 2020. In comparing apparently healthy plants (*S*_0_) of the symptomatic cultivar to asymptomatic cultivars, substantial differences in gas exchange responses and efficiency of the thylakoid reactions were observed. For example, on the initial measurement day, *A*_n_ and *g*_s_ were significantly lower in asymptomatic cultivars compared to the *S*_0_ stage of the susceptible cultivar, whereas *Φ*_PSII_, and ETR were not affected by cultivar in 2019 (Figures 2, 3). Specifically, *A*_n_ (37.6μmolm^−2^ s^−1^) and *g*_s_ (0.82molm^−2^ s^−1^) averaged 14 and 37% lower, respectively, in asymptomatic cultivars relative to *S*_0_ (*A*_n_=43.1μmolm^−2^ s^−1^and *g*_s_=1.31molm^−2^ s^−1^) in 2019. In 2020, *g*_s_ and ETR were significantly lower in asymptomatic cultivar when compared to the *S*_0_ stage of the symptomatic cultivar. For asymptomatic cultivars, *g*_s_ (0.60molm^−2^ s^−1^) and ETR (264μmolm^−2^ s^−1^) were 14 and 11% lower, respectively, than *S*_0_ (*g*_s_=0.70molm^−2^ s^−1^ and ETR=297μmolm^−2^ s^−1^) during the 2020 growing season. While *A*_n_ and *Φ*_PSII_ were statistically equivalent for symptomatic and asymptomatic cultivars in 2020 (Figures 2, 3), the *S*_0_ stage of the symptomatic cultivar had numerically the highest mean values.

Following a period of 3weeks after the initial measurement, diseased plants had progressed through complete necrosis of foliage to sprouting of new leaf tissue. Not surprisingly, *A*_n_, *g*_s_, *Φ*_PSII_, and ETR increased in diseased plants relative to the initial measurement date, but a significant effect of treatment was observed in both years of the study. Among the different stages (*S*_0_–*S*_4_) of CLRDD in the susceptible cultivar, values of *A*_n_, *g*_s_, *Φ*_PSII_, and ETR followed nearly similar trends to the initial measurement date. The values of *A*_n_, *g*_s_, *Φ*_PSII_, and ETR decreased significantly in plants with later stages of CLRDD progression. For example, on the later measurement date, *A*_n_ ranged from 29% lower for *S*_1_ (31.3μmolm^−2^ s^−1^) to 75% lower for *S*_4_ (11.1μmolm^−2^ s^−1^) in 2019 and 49% for *S*_1_ (21.5μmolm^−2^ s^−1^) to 56% for *S*_2_ (19.0μmolm^−2^ s^−1^) lower in 2020, when compared with *S*_0_ (43.9μmolm^−2^ s^−1^ in 2019 and 41.8μmolm^−2^ s^−1^). Similarly, *g*_s_ decreased 40% for *S*_1_ (0.90molm^−2^ s^−1^) to 89% for *S*_4_ (0.16molm^−2^ s^−1^) in 2019 and 46% for *S*_1_ (0.51molm^−2^ s^−1^) to 49% for *S*_3_ (0.49molm^−2^ s^−1^), in 2020, relative to *S*_0_ (1.49molm^−2^ s^−1^ in 2019 and 0.94molm^−2^ s^−1^ in 2020). Declines in Φ_PSII_ and ETR for diseased plants relative to *S*_0_ (*Φ*_PSII_=0.38 and ETR=317μmolm^−2^ s^−1^ in 2019 and *Φ*_PSII_=0.46 and ETR=299μmolm^−2^ s^−1^ in 2020) plants ranged from 27% for *S*_1_ (*Φ*_PSII_=0.27 and ETR=230μmolm^−2^ s^−1^) to 72% for *S*_4_ (*Φ*_PSII_=0.10 and ETR=88μmolm^−2^ s^−1^) in 2019 and from 42% for *S*_1_ (*Φ*_PSII_=0.26 and ETR=173μmolm^−2^ s^−1^) to 59% for *S*_2_ (Φ_PSII_=0.19 and ETR=123μmolm^−2^ s^−1^) in 2020.

**Figure 4:**
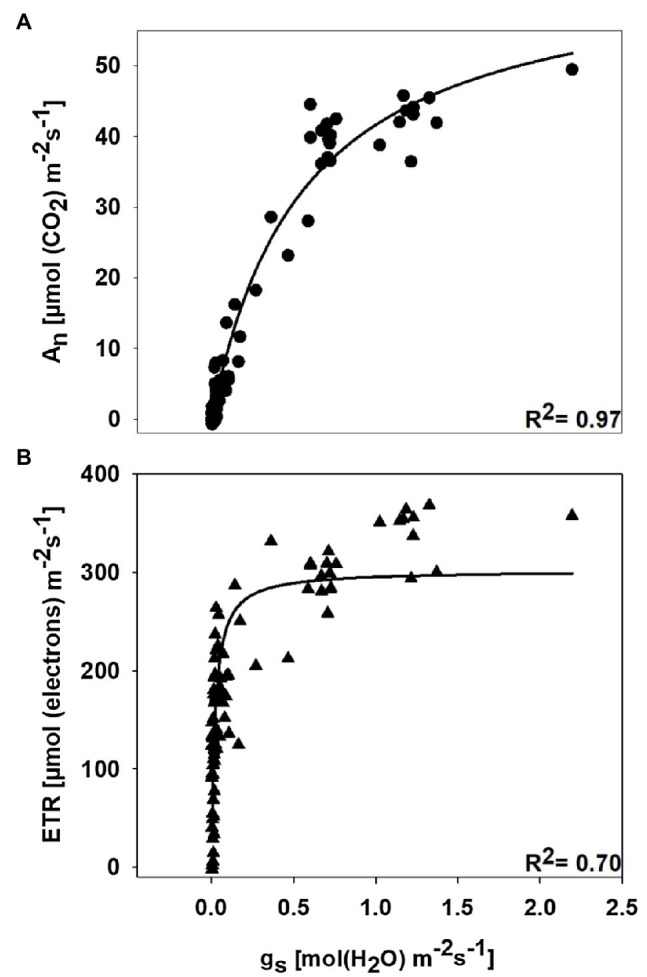
Relationship of net photosynthesis (*A*_n_) with stomatal conductance (*g*_s_; **A**) and relationship of electron flux through photosystem II (ETR) with stomatal conductance (*g*_s_; **B**). Data are from the symptomatic cultivar (DG 3615), at disease stages ranging from *S*_0_ to *S*_4_, and from two growing seasons (2019 and 2020). In both graphs, hyperbolic functions were used for regression analysis.

The relationship between *A*_n_ and *g*_s_ and the relationship between ETR and *g*_s_ are shown in Figure 4. Hyperbolic functions were fit to the observed data, and the regression coefficient for each curve was *r*^2^=0.97 and *r*^2^=0.70 for *A*_n_ vs. *g*_s_ and ETR vs. *g*_s_, respectively. The relative sensitivity of *A*_n_ and ETR to stress was defined by the *g*_s_ value necessary to cause 50% reduction in activity relative to the maximum value observed within the data range. The *g*_s_ value needed to cause 50% decline in *A*_n_ from the maximum was 0.371molm^−2^ s^−1^, whereas the *g*_s_ value causing the same decline in ETR from the maximum was 0.022molm^−2^ s^−1^. This illustrates that *A*_n_ was more sensitive to disease-induced reductions in *g*_s_ than ETR.

### Leaf Temperature and Leaf-Air Temperature Difference

**Figure 5:**
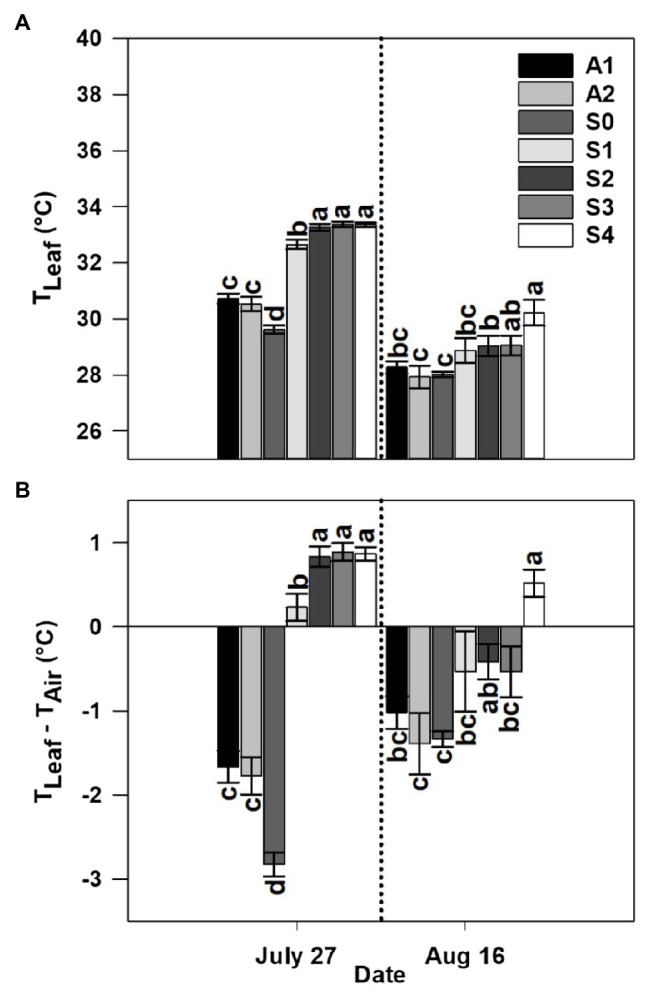
Leaf temperature (*T*_Leaf_; **A**) and leaf-air temperature difference (*T*_Leaf_−*T*_Air_; **B**) for asymptomatic cultivars (*A*_1_ and *A*_2_) and different stages of disease progression in symptomatic cultivars (*S*_0_ through *S*_4_) measured at two different dates in 2019. Error bars indicate standard errors of the mean. *Post hoc* means separation was performed using Fisher’s protected LSD analysis. Within each measurement date, bars with the same letter are not significantly different at *p*≤0.05.

**Figure 6:**
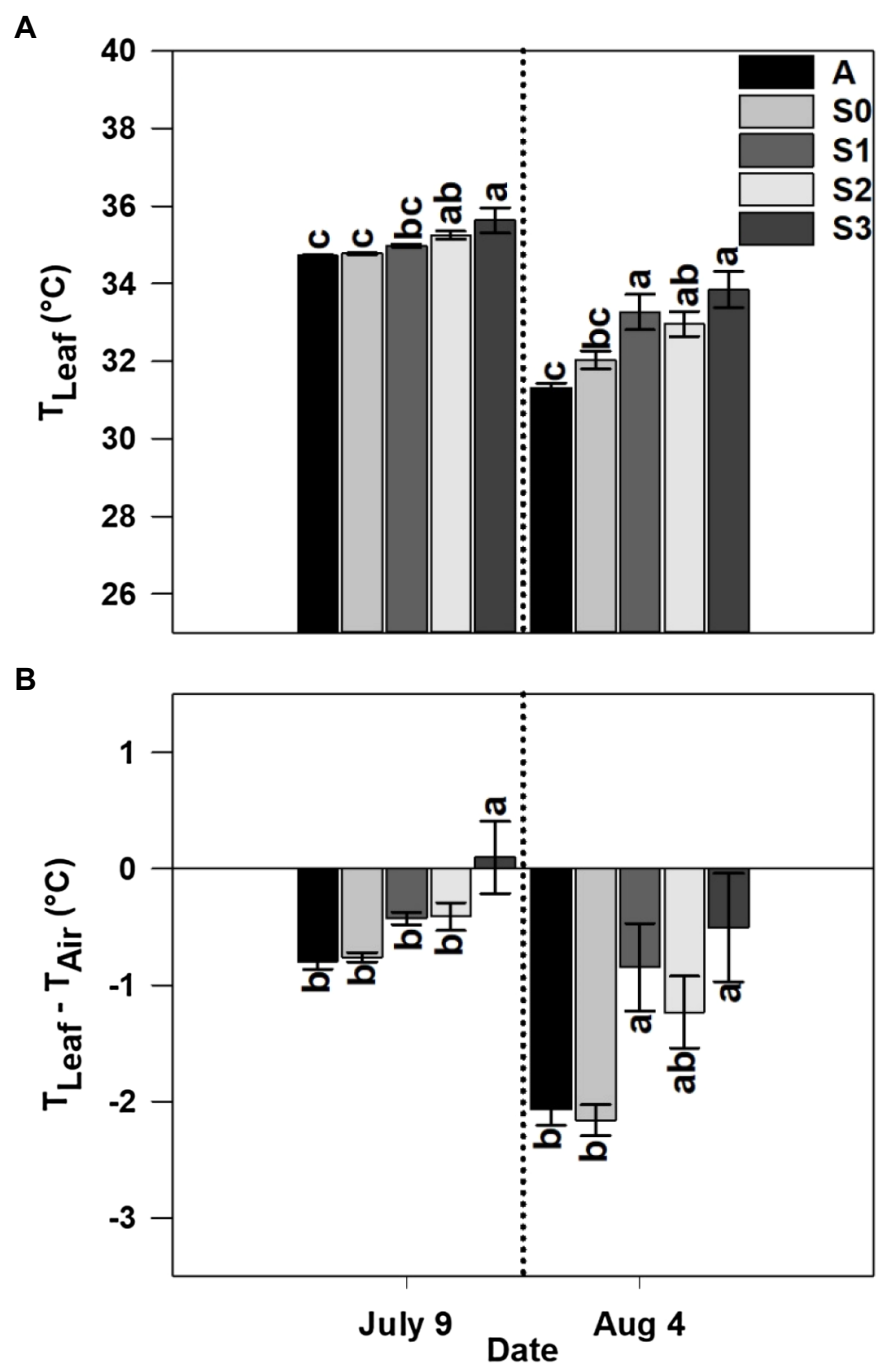
Leaf temperature (*T*_Leaf_; **A**) and leaf-air temperature difference (*T*_Leaf_−*T*_Air_; **B**) for an asymptomatic cultivar (*A*) and different stages of disease progression in symptomatic cultivars (*S*_0_ through *S*_3_) measured at two different dates in 2020. Error bars indicate standard errors of the mean. *Post hoc* means separation was performed using Fisher’s protected LSD analysis. Within each measurement date, bars sharing a common letter are not significantly different at *p*≤0.05.

Leaf temperature was significantly affected by treatment in both years and sample dates within a given year (Figures 5A, 6A). Among the different stages of CLRDD in the susceptible cultivar, leaf temperature increased significantly as the disease progressed to more advanced stages (Figures 5A, 6A). On the first measurement day in 2019, leaf temperature ranged from 0.9°C higher for *S*_1_ leaves to 3.8°C higher for *S*_3_ leaves relative to the *S*_0_ stage. In 2020, leaf temperature was 0.5 and 0.9°C higher in *S*_2_, and *S*_3_, respectively, when compared with *S*_0_. Leaf temperature was not significantly different among asymptomatic cultivars and the *S*_0_ stage of the susceptible cultivars in either year except on July 27, 2019 when asymptomatic cultivars had significantly higher leaf temperature (by 1.1°C in *A*_1_ and 0.9°C in *A*_2_) than the *S*_0_ stage of the susceptible cultivar (Figures 5A, 6A). On the later measurement date in both years, despite production of new leaves following a recovery period, leaf temperature continued to be affected significantly by stage of disease progression (Figures 5A, 6A). Specifically, leaf temperature followed a similar trend as noted on the first sample date, higher leaf temperature with greater disease severity.

Leaf-air temperature difference was significantly affected by treatment, following identical trends to leaf temperature (Figures 5B, 6B). Notably, on the initial measurement date, for all diseased plants, leaf temperature was significantly higher than air temperature 0.86°C higher for *S*_2_–*S*_4_, whereas the *S*_0_ stage was 2.8°C below air temperature on the same date in 2019 (Figures 5B, 6B). In 2020, leaf temperature was lower than air temperature by 0.53°C for *S*_0_–*S*_2_ while leaf temperature for the *S*_3_ stage was higher than air temperature by 0.01°C. Leaf-air temperature difference was not significantly different among asymptomatic cultivars and the *S*_0_ stage of the susceptible cultivar in either year except on July 27, 2019 when the *S*_0_ stage of the susceptible cultivar (−2.8°C) had a significantly more negative leaf-air temperature difference than the asymptomatic cultivars (−1.7°C; Figures 5B, 6B).

### Diurnal Variation in Leaf-Air Temperature Difference

**Figure 7:**
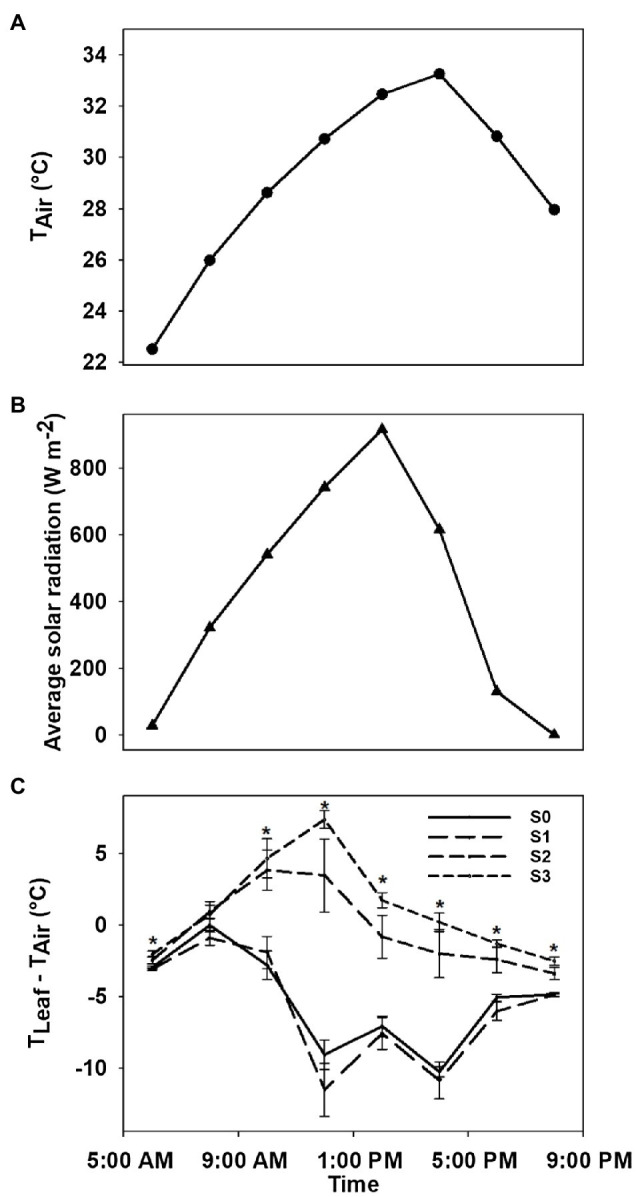
Diurnal variation in air temperature **(A)**, solar radiation **(B)**, and leaf-air temperature difference for plants of a symptomatic cultivar (DG 3615) at different stages of CLRDV disease progression **(C)**. Error bars indicate standard errors of the mean. *Post hoc* means separation was performed using Fisher’s protected LSD analysis. Asterisks indicate a significant effect of disease stage on *T*_leaf_−*T*_air_ at a given time of day.

Diurnal variations in air temperature, solar radiation, and leaf-air temperature difference of plants with different stages of CLRDD are shown in Figure 7. Leaf-air temperature difference was significantly affected by different stages of CLRDD at nearly all sample times throughout the day except at 08:00h when leaf-air temperature difference was the same for all stages. During most of the day, leaf-air temperature was positive for the *S*_2_ and *S*_3_ stages, whereas leaf-air temperature difference was negative for *S*_0_ and *S*_1_, indicating that plants at *S*_0_ and *S*_1_ stages maintained leaf temperatures cooler than air temperature. Leaf temperature exceeded air temperature as much as 3.46°C for *S*_2_ and 7.37°C for *S*_3_ at 12:00h whereas leaf temperature was below the air temperature by 9.08°C for *S*_0_ and 11.52°C for *S*_1_. Relative differences in leaf temperature among different stages started to increase as the day progressed, reached a maximum at 12:00h, and then decreased later in the afternoon. At 12:00h, *S*_2_ and *S*_3_ had average leaf temperatures that were approximately 16°C higher than *S*_0_ and *S*_1_.

### Seed Cotton Yield and Yield Components

Lint yield, seed cotton yield, and all yield components assessed were significantly affected by treatment (Table 2). Among the different stages of CLRDD in the susceptible cultivar, lint yield, and seed cotton yield per plant were decreased by 99% for all diseased plants (*S*_1_, *S*_2_, and *S*_3_) compared to *S*_0_. Boll number per plant showed nearly identical trends to seed cotton and lint yield per plant, and a reduction in boll number was the primary contributor to yield loss in diseased plants. For example, the number of bolls per plant decreased approximately 93% in *S*_1_ through *S*_3_ plants, when compared with *S*_0_ plants. Boll mass and number of seeds per boll were also negatively impacted in diseased plants. Average boll weight decreased by 33% in *S*_1_ plants to 65% in *S*_3_ plants, compared to *S*_0_. *S*_2_ and *S*_3_ plants also averaged nearly 14 fewer seeds per boll than *S*_0_ plants.

**Table 2:** Seed cotton yield, lint yield, and yield components for an asymptomatic cultivar (*A*) and different stages of disease progression in symptomatic cultivars (*S*_0_ through *S*_3_) of cotton in 2020.

Treatment	Seed-cotton yield (g plant^−1^)	Lint yield (g plant^−1^)	Bolls plant^−1^ (no.)	Boll mass (g)	Seed boll^−1^ (no.)
A	45 b	19 b	10 bc	4.39 ab	27 a
S0	93 a	46 a	19 a	4.94 a	26 a
S1	1 c	1 c	2 c	3.29 bc	19 ab
S2	1c	1 c	1 c	2.57 c	15 b
S3	1 c	0 c	1 c	1.74 c	10 b

*Post hoc means separation was performed using Fisher’s protected LSD analysis. Mean values followed by the same letter in each column are not significantly different at p≤0.05*.

Lint yield, seed cotton yield, and number of bolls per plant were lower by approximately half in the asymptomatic cultivar compared to *S*_0_ stage of the susceptible cultivar. In contrast, the number of seeds per boll and average boll weight were statistically equivalent in the asymptomatic cultivar and the *S*_0_ stage of the susceptible cultivar.

## Discussion

In the current study, it was hypothesized that cotton plants of the susceptible cultivar showing CLRDD symptoms would exhibit lower light-saturated photosynthetic rates, reduced photochemical efficiencies of photosystem II, declines in stomatal conductance, increased *T*_leaf_, and reduced productivity relative to plants of the same cultivar without conspicuous disease symptoms. The data presented herein support our hypothesis. For example, net assimilation (*A*_n_), stomatal conductance (*g*_s_), and primary photochemistry (*Φ*_PSII_ and ETR) decreased significantly as CLRDD symptoms progressed to more advanced stages. In fact, *A*_n_ was essentially eliminated in diseased plants for stages *S*_2_ through *S*_4_ on the initial sample date in both years of our experiment (Figures 2, 3). Significant reductions in *A*_n_, *g*_s_, *Φ*_PSII_, and ETR have been reported in viral diseases such as the sugarcane yellow leaf virus (family Solemoviridae, genus *Polerovirus*) in sugarcane (Lehrer and Komor, 2008), grapevine leaf roll-associated viruses (family Closteroviridae, genus *Ampelovirus*), grapevine fan leaf virus (family Comoviridae, genus *Nepovirus*), and grapevine fleck virus (family Tymoviridae, genus *Maculavirus*) in grapevine (Sampol et al., 2003; Bertamini et al., 2004), pepper mild mottle virus and paprika mild mottle virus (family Virgaviridae, genus *Tobamovirus*) in *Nicotiana benthamiana* (Rahoutei et al., 2000), and potato virus A and potato virus Y (family Potyviridae, genus *Potyvirus*) in *N. tabacum*.

In a review by Alazem and Lin (2017), it was noted that ABA content commonly increases in virus-infected plants as a defense response, and increase in ABA in leaves substantially limit *g*_s_. Virus-induced increases in ABA content have been reported for cucumber mosaic virus in *N. benthamiana*, bamboo mosaic virus in *Arabidopsis thaliana*, bamboo mosaic virus in *N. benthamiana*, and tobacco mosaic virus in *N. tabacum* (Fraser and Whenham, 1989; Alazem et al., 2014). Therefore, it is possible that the decline in stomatal conductance that we observed in the symptomatic plants might be due to virus-induced increases in ABA synthesis. Additionally, reductions in *g*_s_ are a well-known response to multiple wilt-inducing stresses (biotic or abiotic), and stomatal closure due to decreases in leaf turgor limits access to CO_2_ by rubisco (Kirkpatrick et al., 1995; Sampol et al., 2003; Farooq et al., 2009; Chastain et al., 2014; Snider et al., 2014; Parkash and Singh, 2020a; Parkash et al., 2021). Furthermore, if *g*_s_ reaches sufficiently low levels, even processes that do not necessarily require CO_2_ as a substrate (e.g., the thylakoid reactions) will become limited (Medrano et al., 2002). Similarly, in our study, *A*_n_ and ETR showed a strong hyperbolic response to *g*_s_ (Figure 4). However, when the relative sensitivity of each process to declines in *g*_s_ is determined, clear differences in stress sensitivity for carbon assimilation and electron transport are observed. For example, the *g*_s_ value required to decrease *A*_n_ 50% from the maximum observed value was 0.371molm^−2^ s^−1^ while a 50% decline in ETR from the maximum observed value was observed at a *g*_s_ value of 0.022molm^−2^ s^−1^. This indicates substantially greater stress sensitivity for carbon assimilation than electron transport. Under other stresses, where electron transport outpaces carbon assimilation (such as drought), oxidative stress is an inevitable consequence (Lawlor, 2002). Furthermore, anthocyanin production under stress is thought to either attenuate excess solar radiation or to serve a reactive oxygen scavenging role (Close and Beadle, 2003). Thus, the possibility that leaf reddening (Figure 1) in the initial stages of CLRDD is a response to oxidative stress should be evaluated in the future. It is well documented that some plant viruses affect the chloroplasts while infesting the host plant (Bhattacharyya and Chakraborty, 2018), altering the expression of chloroplast- and photosynthesis-related genes (CPRGs), and generating disease symptoms in foliage (Bhattacharyya et al., 2015). For example, tobacco mosaic virus causes a reduction in expression of tobacco chloroplast proteins, reducing photosynthetic efficiency (Lehto et al., 2003). Chloroplasts are also a major source for generating reactive oxygen species (ROS; Ambastha et al., 2015), and ROS play a key role in inducing cell death and subsequently causing leaf senescence under various biotic and abiotic stresses (Van Breusegem and Dat, 2006). Overall, this suggests that CLRDV might have interacted with the chloroplast, thereby contributing to lower photosynthesis rate in diseased plants. Moreover, interaction of the virus with the chloroplast might have increased the production of ROS, which subsequently might have caused the cell death and ultimately leaf senescence in diseased plants.

Our data showed that leaf temperature increased as CLRDD symptoms progressed to more advanced stages (Figures 5–7), where leaf temperature of diseased plants was often higher than air temperature. In addition, plants with more advanced stages of CLRDD symptoms had lower *g*_s_, and declines in *g*_s_ limit water loss through the stomatal aperture *via* transpiration. Transpiration is the dominant mechanism by which plants dissipate excess energy and keep foliage cool (Fitter and Hay, 2012). Therefore, declines in *g*_s_ are commonly associated with higher leaf temperature (Chastain et al., 2014; Parkash and Singh, 2020b; Virk et al., 2020a,b; Parkash et al., 2021), as is the case for plants at later stages of CLRDD. Interestingly, while diseased plants have a higher leaf temperature at the hottest part of the day, they also lacked the ability to cool for the majority of the day, even when air temperatures were cooler and solar radiation was lower (e.g., during morning and late afternoon hours). For example, *S*_2_ and *S*_3_ plants had higher leaf temperatures than *S*_1_ or *S*_0_ plants for all sample times after 0900h. Furthermore, at some of the sample times, leaf temperature for *S*_2_ and *S*_3_ plants was at or above air temperature (Figure 7). This is particularly concerning for cotton because the optimal canopy temperature range for metabolic activity is 28±3°C (Burke and Wanjura, 2010). Without the ability to maintain a limited level of homeothermy through transpiration (Mahan and Upchurch, 1988), the effects of plant disease on metabolism are likely only magnified by concurrent exposure to heat stress.

We noted that symptoms of CLRDD described herein (leaf reddening, drooping, increased leaf temperature, and wilting) are also symptoms commonly indicative of bronze wilt (Bell et al., 2002). However, symptoms of bronze wilt often appear during fruit development and no pathogen has been identified as the causal agent; therefore, it was believed to be a physiological disorder associated with specific cotton germplasm tracing to the genetic background of “Tamcot SP-37.” Bronze wilt was particularly prevalent in the 1990s, but the removal of susceptible varieties from the market and avoiding the use of susceptible germplasm in breeding programs have largely eliminated the disease in the United States cotton belt. Nonetheless, it is an insidious disease with an occasional susceptible variety making its way to the market, resulting in localized outbreaks. Because of the substantial overlap in symptomology between bronze wilt and CLRDD, future studies should determine if the two diseases share a common cause.

Plants expressing disease symptoms in the early season stayed extremely stunted throughout the remainder of the growing season, which ultimately limited plant productivity. For example, per plant lint and seed-cotton yield was almost completely eliminated in plants expressing all disease stages (*S*_1_–*S*_3_) in the 2020 season. Among the yield components assessed in the current study, the primary driver of yield loss in diseased plants was a reduction in the number of bolls per plant. Specifically, conspicuously-diseased plants only retained an average of 1–2 bolls per plant, whereas *S*_0_ plants set an average of 19 bolls per plant (Table 2). This observation is in agreement with a recent report that severe disease incidence results in reduced boll set and ultimately reduces seed-cotton yield per plant (Avelar et al., 2020). Field observations of diseased plants suggest that pollen development or shedding may have been compromised but this requires further study. In addition to reductions in boll number, the average boll weight and seed set per boll were also significantly reduced, likely contributing to reductions in per plant lint and seed-cotton yield. Cotton yield losses up to 80% (per unit land area) have been reported due to Blue disease caused by the CLRDV biotype prevalent to South America (Silva et al., 2008). In the current study, seed cotton yield per plant decreased by 99%. Therefore, the future impact of the recently detected North American CLRDV genotype on the United States cotton industry will largely depend on the development of resistant varieties, and if susceptible varieties are planted, the levels of disease incidence which can be attenuated by controlling the cotton aphid vector.

In the current experiment, we also hypothesized that asymptomatic cultivars would exhibit more conservative gas exchange responses (lower *g*_s_) than otherwise healthy-appearing plants of the symptomatic cultivar under field conditions. Our results tentatively support this hypothesis. For example, when evaluated under the same mid-day conditions in 2019, plants of the symptomatic cultivar in the *S*_0_ stage had higher stomatal conductance (*g*_s_) and net photosynthesis (*A*_n_) than either asymptomatic cultivar on the initial measurement date. In 2020, the *S*_0_ stage was found to have significantly higher *g*_s_ and numerically higher *A*_n_ than an asymptomatic cultivar. There are two possible explanations for our observations. First, healthy plants of the symptomatic cultivar possess innately higher gas exchange rates than asymptomatic cultivars, even in the absence of CLRDV. Recent research has indicated that cotton cultivars exhibit genotypic differences in their gas exchange response to vapor pressure deficit (VPD; Shekoofa et al., 2021). For example, VPD will increase as air temperature increases or relative humidity decreases. Some cultivars will close their stomata to limit gas exchange at high VPD to conserve water and delay wilting. Other cultivars maintain higher gas exchange rates even under hot, dry conditions, making them more susceptible to rapid-onset wilting. Since all plants (normal or with conspicuous disease symptoms) tested positive for the presence of the virus, it was not possible to distinguish these two possibilities. Additionally, the S_0_ stage of symptomatic cultivar had higher per plant yields than the asymptomatic cultivar. Thus, in the absence of disease symptoms, higher gas exchange rates in the susceptible cultivar may promote higher yields when compared with asymptomatic cultivars that have more conservative gas exchange rates.

The second possible explanation for our observation is that viral infection induces higher gas exchange rates in otherwise healthy plants by increasing *g*_s_, but only in susceptible cultivars. In a study on potato, it was found that a negative control and virus (potato virus Y) infested plants had similar *g*_s_ initially but it decreased in virus infested plants over time (Zhou et al., 2004). Though not due to viral infection, in two rice genotypes, initially *g*_s_ increased after pathogen infestation (bacterial blight) then decreased with time in pathogen inoculated plants compared to the negative control, whereas a third genotype had lower *g*_s_ in pathogen infested plants during all sampling times after pathogen infestation (Kumar et al., 2013). To elucidate interactions between CLRDV and cultivar for physiological responses to infection will require controlled field experiments in the future that incorporate negative and positive control plants of symptomatic and asymptomatic cultivars.

## Conclusion

The objectives of the current study were to determine gas exchange, chlorophyll fluorescence, and leaf temperature responses for a symptomatic cotton cultivar at multiple stages of CLRDD progression and for otherwise healthy-appearing plants of symptomatic and asymptomatic cultivars. The results of our study suggest that symptomatic cultivars exhibiting CLRDD had significant declines in *g*_s_, net photosynthetic rate, and photochemical efficiency of the thylakoid reactions. Photosynthetic electron transport was less sensitive to declines in *g*_s_ than was carbon assimilation, and future research should evaluate the possibility that an imbalance between the thylakoid reactions and carbon assimilation leads to oxidative stress in diseased tissues. Another consequence of lower *g*_s_ associated with CLRDD was a reduction in the ability to cool through transpiration. This was evidenced by higher leaf temperatures and positive leaf-air temperature differentials for much of the day. Ultimately, the negative impacts of CLRDD on plant physiological processes resulted in near-complete loss of per plant yield in diseased plants. Disease-free plants of the symptomatic cultivar exhibited higher gas exchange rates than the asymptomatic cultivar and had greater per plant productivity. The possibility that conservative gas exchange responses in asymptomatic cultivars promote tolerance to CLRDV should be evaluated further in controlled field experiments. Finally, the relationship between bronze wilt and CLRDD should be investigated.

## Data Availability Statement

The original contributions presented in the study are included in the article/supplementary material, and further inquiries can be directed to the corresponding authors.

## Author Contributions

JS, PC, DS, and SB contributed to the conception and design of the study. JS, PR, DW, SK, and NS were responsible for project execution. VP analyzed the data and wrote the manuscript. All authors contributed to the article and approved the submitted version.

## Funding

This research was funded in part by Cotton Incorporated (grant number 19-114) and by Georgia Cotton Commission.

## Conflict of Interest

The authors declare that the research was conducted in the absence of any commercial or financial relationships that could be construed as a potential conflict of interest.

## Publisher’s Note

All claims expressed in this article are solely those of the authors and do not necessarily represent those of their affiliated organizations, or those of the publisher, the editors and the reviewers. Any product that may be evaluated in this article, or claim that may be made by its manufacturer, is not guaranteed or endorsed by the publisher.
